# Supplementary material for: Splice-Junction-Based Mapping of Alternative Isoforms in the Human Proteome
Source: Cell Rep. Author manuscript; Available in PMC 2020 Jan 15. (PMC6961840; doi:10.1016/j.celrep.2019.11.026)
Supplement: 3 [file NIHMS1546469-supplement-3.zip › DF2/PXD000561/Liver-37-Q13438-KPPPSPQPTGK.pdf]

A

Predicted sequence disorder and sequence features of Q13438

Peptide: KPPPSPQPTGK Junction: sp|Q13438|OS9_HUMAN|ENSG00000135506|SE2|9890|chr12|57719182|57720263|+0|r93|T1 TrNovel: FALSE

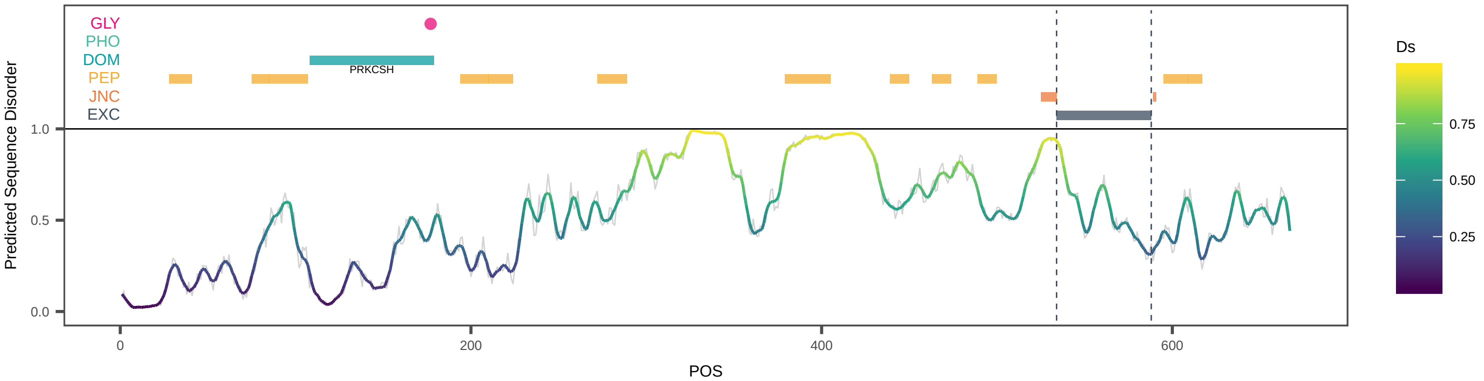

B

Distribution of sequence disorder in excised
vs. mapped and non−excised regions of protein

M−W P−value vs. mapped: 0.182 vs. non−excised: 0.475

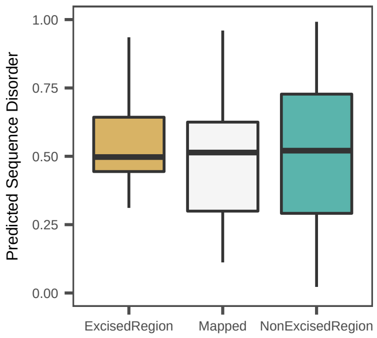

C
